# Healthy Choice Rewards: A Feasibility Trial of Incentives to Influence Consumer Food Choices in a Remote Australian Aboriginal Community

**DOI:** 10.3390/ijerph16010112

**Published:** 2019-01-03

**Authors:** Clare Brown, Cara Laws, Dympna Leonard, Sandy Campbell, Lea Merone, Melinda Hammond, Kani Thompson, Karla Canuto, Julie Brimblecombe

**Affiliations:** 1 Apunipima Cape York Health Council, 4870 Cairns, Australia; cara.laws@apunipima.org.au (C.L.); lea.merone@apunipima.org.au (L.M.); melinda.hammond@apunipima.org.au (M.H.); kani.thompson@apunipima.org.au (K.T.); karla.canuto@jcu.edu.au (K.C.); 2 Australian Institute of Tropical Health and Medicine, College of Public Health Medical and Veterinary Sciences, James Cook University, 4870 Cairns, Australia; dympna.leonard@jcu.edu.au; 3 Centre for Indigenous Health Equity Research, Central Queensland University, 4870 Cairns, Australia; s.campbell@cqu.edu.au; 4 Wardliparingga Aboriginal Health, South Australian Health and Medical Research Institute, 5001 Adelaide, Australia; 5 Department of Nutrition, Dietetics and Food, Monash University, 3168 Melbourne, Australia; julie.brimblecombe@monash.edu

**Keywords:** Aboriginal and Torres Strait Islander, remote, community store, fruit and vegetables, incentive, subsidy, food security, nutrition, diet

## Abstract

Poor diet including inadequate fruit and vegetable consumption is a major contributor to the global burden of disease. Aboriginal and Torres Strait Islander Australians experience a disproportionate level of preventable chronic disease and successful strategies to support Aboriginal and Torres Strait Islander people living in remote areas to consume more fruit and vegetables can help address health disadvantage. Healthy Choice Rewards was a mixed methods study to investigate the feasibility of a monetary incentive: store vouchers, to promote fruit and vegetable purchasing in a remote Australian Aboriginal community. Multiple challenges were identified in implementation, including limited nutrition workforce. Challenges related to the community store included frequent store closures and amended trading times, staffing issues and poor infrastructure to support fruit and vegetable promotion. No statistically significant increases in fruit or vegetable purchases were observed in the short time frame of this study. Despite this, community members reported high acceptability of the program, especially for women with children. Optimal implementation including, sufficient time and funding resources, with consideration of the most vulnerable could go some way to addressing inequities in food affordability for remote community residents.

## 1. Introduction

Food security is a major global issue [1]. Strategies to achieve physical and economic access to sufficient, safe and nutritious food are important for all and this is especially important for Indigenous Peoples who often experience the most severe economic and health disparities [2]. Australia is a wealthy country but high levels of food insecurity have been documented for Aboriginal and Torres Strait Islander people compared to other Australians (22% versus. 3.7%) [3]. In Australia, food insecurity is highest among Aboriginal and Torres Strait Islander people living in remote locations (31%) compared to non-remote (20%) [3].

The life expectancy gap of 10–11 years between Aboriginal and Torres Strait Islander people and non-Indigenous Australians is well known [4]. Recent national survey reports indicate that Aboriginal and Torres Strait Islander people consume a diet that is relatively poor compared to other Australians, with lower intakes of fruit and vegetables and higher intakes of sugar sweetened beverages and nutritionally poor foods [3]. Chronic diseases, much of which are diet-related were responsible for 70% of the gap in health between Aboriginal and Torres Strait Islanders and non-Indigenous Australians in 2011 [5]. Contributing to this are the higher rates of overweight and obesity, cardiovascular disease, chronic kidney disease and type two diabetes [4]. 

Many factors influence the nutritional status of Aboriginal and Torres Strait islander people, including socioeconomic disadvantage and other historical, social, environmental and geographical factors [6,7,8]. Healthy foods in remote Aboriginal and Torres Strait Islander communities cost more than urban areas [9,10,11]. The 2016 Census shows the median household weekly income in the remote region of interest is AUD $987 for a mean household size of 3.8 people [12]. This is 70% of the median state of Queensland household income of AUD $1402 per week with a lower average household size of 2.6 people [12]. On this lower income, remote area residents in Queensland pay 41% more for fruit and 12% more for vegetables compared with Queenslanders living in urban areas [9]. Research has shown that when food choices are made under budget constraints, consumer purchasing behaviour is driven by maximising energy value for money (dollars per megajoule), resulting in the purchase of fewer nutrient rich foods such as fruit and vegetables and more nutrient poor, energy dense foods [13,14].

There is a well-established link between increased fruit and vegetable consumption and improved health outcomes [15]; consequently, increasing consumption of fruit and vegetables has been identified as an important measure to achieve health gains nationally [16]. In addition to improved health, it has been estimated that if vegetable consumption in Australia was 10% higher, government expenditure on health care could be reduced by AUD $100 million annually [17]. If all Australians met the recommended daily intake of vegetables this saving would increase nine fold [17]. The potential savings are likely to be more pronounced for Aboriginal and Torres Strait Islander people living in remote areas due to the higher burden of disease experienced and the high costs of delivering remote health services.

In the context of increasing health care costs and government budget cuts threatening progress in the prevention of chronic disease [18], it is important to investigate cost effective measures to address health disadvantage for Aboriginal and Torres Strait Islander people living in remote communities and provide clear recommendations for policy makers. There is a growing body of research demonstrating the potential for food price changes to influence diet quality and drive positive population health gains [19,20,21,22,23,24]. Government policy options in pricing strategies include unhealthy food taxation, healthy food subsidies and price discount schemes to promote healthy food environments [25,26]. In Australia, two large supermarket price discount randomised controlled trials have recently been completed, both showing the effectiveness of price discounts on fruit and vegetable purchasing [22,23]. One of these projects, the Stores Healthy Options at Remote Indigenous Communities (SHOP@RIC) was implemented in 20 remote communities in Northern Territory and achieved a 12.7% increase in purchases of fruit and vegetables [22].

Strategies to make fruit and vegetables more accessible to Aboriginal and Torres Strait Islander families living in remote communities have the potential to reduce health inequality and subsequent health care costs. Here we report on a feasibility trial of a monetary incentive to promote fruit and vegetable purchasing in one remote community. To our knowledge, the effectiveness of immediately rewarding healthy purchasing behaviours has not yet been explored in a remote Aboriginal and Torres Strait Islander community context. 

## 2. Materials and Methods

### 2.1. Setting

Apunipima Cape York Health Council (Apunipima) community nutrition project staff conducted a study in 2015 to assess the feasibility of implementing a fruit and vegetable incentive in a very remote Australian community store in far north Queensland, located around 2500 km from the nearest major city. This remote community has approximately 1400 residents with most (90.4%) identifying as Aboriginal and/or Torres Strait Islander [27]. The community experiences low levels of formal education, low income, reliance on social security payments and high dependency ratios [27]. While the people of this community value traditional foods and traditional food systems, the community store is the main source of food for residents for their daily needs. The next nearest grocery store is 200 km away; a three hour drive by dirt road. 

### 2.2. Design

A mixed methods approach was used and included collection of qualitative data using semi-structured interviews, participant observation, a weekly electronic survey on store and wider community contextual information and a quantitative assessment of store sales data. Feasibility of the intervention was assessed in terms of acceptability, voucher uptake, implementation issues and impact on fruit and vegetable sales. All customers of the store were eligible to participate. Study implementation was led by Apunipima community nutrition staff. 

### 2.3. Healthy Choice Rewards Program 

The Healthy Choice Rewards (HCR) program offered community store customers an incentive of a fruit and vegetable voucher to the value of AUD $10 each time a set minimum amount was spent on fruit and vegetables. Store staff participated in semi-structured interviews prior to the study to inform the reward system design and determine what supports would need to be in place for implementation. Two phases of the minimal amount spent were trialed: phase one required a AUD $20 spend on fresh fruit and vegetables to receive a AUD $10 HCR voucher to be redeemed on the date of purchase; phase two required a AUD $15 spend on fresh fruit and vegetables to receive a AUD $10 HCR voucher to be redeemed within three days. Frozen, tinned and dried fruit and vegetables were not included as part of the minimum spend as they could not be easily distinguished in the store’s electronic grocery management system. The vouchers were redeemable for fresh, frozen, tinned or dried fruit and vegetables and excluded tinned fruit in syrup and frozen potato chips and wedges. Vegetable packs valued at AUD $10 were available for sale. The store was reimbursed for the value of any vouchers used. 

The incentive was available for 32 weeks; phase one ran for 15 weeks, followed immediately by phase two for 17 weeks. The HCR vouchers appeared as black and white plain text print outs at the end of customer store dockets. The reward offer was promoted in English and local language using posters, flyers, radio advertisements and electronic register screen displays at the store.

Project staff visited the community monthly during the intervention period to promote HCR. During the visits they delivered healthy cooking demonstrations, distributed healthy recipe flyers, spoke with community members on how to utilise the offer and assisted store staff in merchandising the fruit and vegetable display. Between visits the project team provided weekly phone and email support to the store manager to maintain program promotion and assist with processing the vouchers. 

### 2.4. Data Collection

To determine the feasibility of the HCR program the primary outcome measures included: acceptability of the voucher incentive to customers and store staff, voucher uptake and redemption and identification of the opportunities and challenges of implementation. A secondary measure included per capita total fruit and vegetable intake derived from store sales purchasing data. 

#### 2.4.1. Acceptability 

Following completion of both phases of the intervention, we invited store staff and store customers (community members) to provide feedback through semi-structured face-to-face interviews (customer or staff satisfaction interviews). Demographics on age, gender, Aboriginal and Torres Strait Islander status, and employment were collected. Project staff (one of whom is a Torres Strait Islander woman) with training and experience interviewing Aboriginal and Torres Strait Islander community members conducted the interviews. To promote the feedback opportunities to the community, the team engaged in local activities including performing a healthy cooking demonstration and organising a group fishing trip with a healthy lunch. All customer and store staff interviewees (*n* = 34) received a fruit and vegetable voucher to the value of AUD $10 to acknowledge their time contributed. In addition to the customer satisfaction interviews conducted at the completion of the program, four customer interviews were conducted during phase one to inform intervention changes for phase two. 

#### 2.4.2. Voucher Uptake 

Weekly HCR voucher redemption data were collected using the stores’ electronic point of sale system.

#### 2.4.3. Implementation 

Interview data was also used to assess implementation issues. Project staff also routinely recorded observational data with hand written notes on their regular community visits.

#### 2.4.4. Fruit and Vegetable Sales

Electronic point-of sale data including product description, unit weight, number of units sold, and dollar value were collected weekly for all food and drink sales for the duration of the project period and the same time-period in the previous year. A purpose built weekly electronic survey used in the SHOP@RIC trial [22] collected descriptive data from the store manager on potential factors influencing usual food and drink purchasing such as population movements, community events and activities, frequency of food delivery to the store and retail management practices. This data collection aimed to contextualise store sales data and account for community-level factors that may have influenced purchasing behaviours during the intervention.

### 2.5. Data Analysis

#### 2.5.1. Acceptability and Implementation

Interview data and project observations were collated in Excel. Two project staff members independently coded interview responses and grouped these into emerging themes. Apunipima staff members who had research experience reviewed the coding results and resolved inconsistencies by consensus.

#### 2.5.2. Voucher Uptake

To evaluate HCR voucher uptake, we compared the number of vouchers issued and number of vouchers redeemed across both program phases. 

#### 2.5.3. Fruit and Vegetable Sales

A pre-post point-of-sale analysis of purchasing was completed by Menzies School of Health Research in January 2016. Weekly point-of-sale data were uploaded to a purpose-built Microsoft Access database and coded into relevant food groups. Aggregated weekly point-of-sale data for the 32 week study period were compared to the same time-period in the previous year, to account for seasonal variation. Per capita daily fruit and vegetable consumption for the community was estimated by dividing the average sales for the study period by 32 weeks × 7 days and the usual population estimates obtained from 2011 Australian Bureau of Statistics national census data. The average per capita daily amounts of fruit and vegetables purchased were converted from weights measured in grams to average number of serves using Australian Dietary Guideline definitions for standard fruit and vegetable weights (i.e., 150 g per serve for fruit and 75 g per serve for vegetables) [16]. Statistical analysis was performed using the paired t-test technique to compare sales in phase 1 and phase 2 with the baseline time-periods in the previous year. *p* Values of less than 0.05 were considered statistically significant. Contextual factors were uploaded into an Access Database and frequency of occurrence was graphed on a weekly timescale and considered against the results reported from the purchase data to identify potential variables impacting store sales and to assist interpretation of the impact of the incentive on the outcome measures. 

### 2.6. Ethical Approval

James Cook University Human Research Ethics Committee (HREC H5938) and the combined Northern Territory Department of Health and Menzies School of Health research Human Research Ethics Committee (HREC 2014-2313) granted ethical approval for the study.

## 3. Results

### 3.1. Acceptability

A total of 28 post program customer satisfaction interviews were completed. The majority of customers interviewed identified as Aboriginal and/or Torres Strait Islander people (82.1%) and were women (71.4%). Additionally, 68% of responders reported being employed at the time of the interview. Of those interviewed, more women that were employed than not employed reported using the HCR voucher. All respondents reported they would like the offer to continue and 61% of respondents indicated that HCR encouraged their family to consume more fruit and vegetables. All store staff interviewed following the completion of the project (*n* = 6) reported they wanted the offer to occur again. Community members identified that healthy eating was important for health but there were many challenges and competing priorities to eat a healthy and nutritious diet. They also provided suggestions for improving the program.

#### 3.1.1. Community Perceptions of Healthy Eating 

Healthy eating was viewed as important for participants and HCR was seen as a valuable program as it promoted healthy food choices, as one grandmother said, “It’s important, it is very important. Kids need to eat healthy. We don’t want to see our kids wither away, we want them to have a healthy choice” 

The HCR program was also seen as a good reminder to consume fruit and vegetables, “Like you remind kids, ‘don’t do that’, it’s good to remind us Aboriginal people to eat more fruits and vegetables because sometimes we forget” [Female Elder]. 

Healthy eating was seen as especially important for women with children or young families, as one female participant responded, “Being healthy is especially important for kids to grow strong, good clean blood for [to prevent] anaemia… [it is] good for people with plenty of children, good for their health”. 

#### 3.1.2. Challenges and Competing Priorities to Consume a Healthy Diet 

Community members described facing many challenges to healthy eating including high food costs and limited available money to spend on healthy food, as one participant described, “It’s expensive here, there is hardly enough money to buy food”.

Although the HCR program was valued and seen to encourage fruit and vegetable consumption, it was not enough to alleviate the high cost of food as one respondent indicated, “It was really good. It encourages people to get more fruit and vegetables. AUD $10 doesn’t get you much, but it’s good”. 

While another participant reported, “AUD $10 only gets you two or three fruit and vegetables because of costings of the shop”. 

Other reported challenges to consuming healthy eating included limited access to health hardware such as no fridge to store food at home, limited availability of fresh produce and concerns over quality of this produce by the time it arrives in the community. It was also noted that community members have increasing dependency on takeaway foods rather than preparing homemade meals. Another concern was that children are now preferring the taste of sweet discretionary foods from the store rather than traditional bush foods. 

Some responders also reported that healthy eating was not a priority for everyone, for example there may be other competing priorities based on social factors that are viewed as more important or there may be basic challenges such as the inability to shop for groceries. This was particularly thought to be the case for people with little money, those who did not live at one fixed address (living between multiple houses), those relying on meals provided by family (such as the frail elderly) and even people who struggled with addictions such as alcohol, drugs or gambling. 

#### 3.1.3. Suggestions for Improving the HCR Program 

Feedback from the customer interviews suggested that future incentives may be more effective if the reward system was tailored specifically for women with children and used electronic store loyalty cards instead of paper-based vouchers. Other recommendations from the interviews included: increased flexibility of redemption parameters, more support from store staff (such as explaining the voucher and helping determine AUD $10 worth of fruit and vegetables so it is more convenient for the customer), offering higher incentives and strengthening promotion through increased community involvement. Store staff observed an increase in customer interest in HCR following promotion by the visiting nutrition team and noted that customers reported that uptake of the incentive could have been be improved with greater promotion. 

### 3.2. Voucher Uptake

Voucher redemption rates averaged 28.6% (95% CI: 26, 31) for the duration of the study. A total of 2150 vouchers were issued and 632 redeemed. Redemption rates were higher during phase two of the study compared to phase one, averaging 30% (95% CI: 30, 31) and 27% (95% CI: 21, 32) respectively. The highest redemption rate (44%) was recorded on a week when project staff were at the store performing cooking demonstrations raising awareness about the project and assisting with merchandising of fresh produce.

### 3.3. Implementation 

Four of the six staff interviewed reported having issues with the reward offer and required more support to run the offer. Issues identified by store staff included: being unsure of how to process the voucher in the store electronic grocery management system; having too many customers at once to help other customers claim their voucher; limited time to prepare the AUD $10 fruit and vegetable packs for customers to redeem; customers complaining of losing their receipts and customers refusing the voucher as it meant they needed to queue up a second time to redeem their reward.

Several challenges that impacted on project implementation at the store level were observed by project staff including store infrastructure issues; support for store staff to run the offer; and support for store managers to promote fresh produce. Fresh produce displays were impacted by transport issues; infrastructure issues, such as limited equipment to display produce; the hot climate affecting the temperature control of open display refrigeration units; and store air-conditioning and refrigeration units often breaking down. Supporting store staff proved challenging due to a shortage of trained and experienced staff; high turnover and low attendance among store staff; variable expertise among store management in merchandising of fresh produce and limited capacity of Community and Store Nutritionists to provide sufficient support to store staff. In addition, due to issues impacting the community during the study period such as community unrest, forced store closures and amended trading hours were reported on 23 out of 32 weeks.

Implementation of the project was strengthened by the existing rapport of project staff with community and regular presence in the community; strong partnerships with industry and the research sector; and support from community, local Council and the Store Group to implement the project. 

### 3.4. Fruit and Vegetable Sales 

The voucher incentive was not successful in increasing fruit and vegetable store sales during the study period compared to sales for the same time period in the previous year. In fact, despite including voucher purchases, a 7% reduction in total fruit sales was observed between the two periods, decreasing from 41 to 38 g/person/day (0.27 to 0.25 serves/person/per day), *p* = 0.01. Non-significant reductions in sales of vegetables and overall food and drink sales were also observed. 

## 4. Discussion

This feasibility study describes a monetary incentive strategy to promote fruit and vegetable consumption in a remote Aboriginal community in Australia. While we were unsuccessful in increasing fruit and vegetable purchases during the intervention period, qualitative data indicates that there was a high level of acceptability of the program by community members. This study also highlights the many challenges to be considered in implementing food subsidy strategies to improve nutritional health in the remote community context. 

The HCR project was completed in 2015 as part of implementing a key objective of the Cape York Food and Nutrition Strategy—to ensure equitable food affordability, availability and access comparable to urban Australia [28]. This project therefore works towards addressing the high costs of nutritious foods in remote Aboriginal and Torres Strait Islander communities; a known barrier to healthy eating. 

Although the project staff made frequent trips to the community, store staff and management identified the need for more support. Furthermore, interview data suggested that voucher uptake could have been improved with strengthened promotion. These findings are consistent with other studies demonstrating that consumers need to be made aware of promotional offers [29]. Limited funding for this project and limited community nutrition workforce on the ground restricted promotion efforts. Sufficient resource allocation for promotion and nutrition workforce should be prioritised in future programs. Additionally, interview data indicated that the paper-based voucher system was not always well understood by customers and was reported to be a barrier to participation and could have therefore influenced voucher uptake. An electronic store loyalty card system was recommended by stakeholders as a preferred alternative. This option was explored in the early phases of study, however, the cost of implementing the system with such limited funding was prohibitive but should be considered in any future interventions. 

Women who were employed were most likely to report using the HCR in customer satisfaction interviews. Qualitative data indicated that healthy eating was considered by community members to be more of a priority for women with children or young families. Given that improving access to nutritious food for at-risk mothers, infants and children is a key priority of the 2013–2023 National Aboriginal and Torres Strait Islander Health Plan [30], these findings warrant further investigation. If a reward incentive or subsidy were to be targeted towards smaller population subgroups such as women with children, an individual or household level measure of food and drink purchasing would be needed rather than store population level purchasing data.

This study provided information of fruit and vegetable consumption data for this community which differ from other information sources. Average fruit and vegetables sales were estimated to be equivalent to 0.25 serves/person/day for fruit and 0.92 serves/person/day for vegetables during the study period. These results are lower than self-reported data from the 2012–2013 National Aboriginal and Torres Strait Islander Nutrition and Physical Activity Survey (NATSINPAS) which reported Aboriginal and Torres Strait Islander people living in remote areas across Australia consume on average 0.9 serves of fruit and 1.7 serves of vegetables per day [31]. The NATSINPAS combines results from remote and very remote areas and includes fruit and vegetable components from mixed food sources (such as lasagna), which will likely result in a higher reported intake [31]. While observed differences may also be the result of the different methodology used, a recent comparison of dietary estimates from the very remote sample of the NATSINPAS to food and beverage purchase data from 20 remote Northern Territory community stores suggests over-reporting of fruit and vegetable consumption with self-reporting data [32]. A strength of using sales data is that in a very remote community where there is only one food retail store it provides an objective proxy of population diet [32,33]. For this study, the closest alternative food retail store is 200 km away from the community. Our results are more consistent with a Northern Territory study which reported an average of 0.3–0.7 serves of fruit and 1.1–2.1 serves of vegetables sold per person per day across three remote Aboriginal communities [34].

The limitations of this study are that it was conducted in one remote community only and for a short time period, with limited staffing. These factors reflect the currently limited resources available for nutrition promotion in this setting, compared to previous investments [35]. With additional resources, more support could be provided to the store for implementation, and other factors contributing to the low uptake of the vouchers and reductions on sales of fruit and vegetables could be clarified and addressed. It is likely however that the issues impacting the community at the time which resulted in a high number of forced store closures and amended trading hours influenced voucher uptake and purchases of fruit and vegetables. A strength of HCR was that the voucher incentive was well received by community members and the majority of participants in the evaluation indicated that it helped their family to consume more fruit and vegetables. It was particularly seen as important for mothers with children who needed fruit and vegetables for a healthy start in life. 

Another strength of this project was the strong partnerships and relationships formed by the project team with the community, particularly with the local community store as HCR was supported by store managers, staff and at management levels of the store group. Store Managers play an important role in food supply in remote Aboriginal and Torres Strait Islander communities and are therefore essential partners in helping to improve dietary intake [36]. This project illustrates how the store managers can be effectively supported by nutritionists to actively promote the incentive, resulting in increased uptake.

Remote community stores have an important influence on community health through their ability to control the availability and accessibility of both healthy and unhealthy foods [36]. Significant store implementation challenges were observed in this study. This highlighted the difficulties remote retailers face in maintaining normal store operations, in addition to the ability to adequately support health promotion efforts. Investing in assistance for remote retailers to provide healthy foods to communities is critical to the success of any efforts to improve fruit and vegetable purchase and consumption in remote Aboriginal and Torres Strait Islander communities. 

## 5. Conclusions

This mixed methods feasibility study showed high levels of acceptability of the program by community. It also resulted in the identification of several challenges to be considered when implementing a food subsidy strategy or incentive in remote Australia. Investing in remote retailers to overcome the challenges in providing healthy foods is critical to the success of any efforts to improve fruit and vegetable consumption in remote Aboriginal and Torres Strait Islander communities. Additionally, increased investment in a nutrition prevention workforce to implement healthy remote store practices and support retailers to promote nutrition is required. 

Feedback from customer interviews suggested that future incentives may be more effective if the reward program was tailored specifically for women with children. A larger scale controlled study targeting women and children may provide greater insight into the use and appropriateness of a fruit and vegetable subsidy in the remote community context. 

Consumer food subsidy schemes can help overcome financial barriers and increase affordability of healthy food and drink in remote areas. The high rates of food insecurity in remote Aboriginal and Torres Strait Islander communities are largely a consequence of high rates of unemployment and low incomes compounded by high food costs. Government commitment is needed to reduce the underlying social inequality and to address the affordability of healthy food choices to help close the gap in Aboriginal and Torres Strait Islander health.
